# Analytical and clinical agreement between the Siemens Immulite 2000 veterinary cortisol and standard cortisol assays in canine serum

**DOI:** 10.1093/jvimsj/aalag105

**Published:** 2026-06-05

**Authors:** Ioannis L Oikonomidis, Charalampos Attipa, Aurora Iseberi, Marilena Chatzistylianou, Theodora K Tsouloufi

**Affiliations:** Department of Veterinary Anatomy, Physiology and Pathology, Institute of Infection, Veterinary and Ecological Sciences, University of Liverpool, Neston, United Kingdom; VetPath Specialists, Attica, Greece; VetPath Specialists, Attica, Greece; The Royal (Dick) School of Veterinary Studies, The University of Edinburgh and The Roslin Institute, Midlothian, United Kingdom; Centre for Inflammation Research, Institute for Regeneration and Repair, The University of Edinburgh, Edinburgh, United Kingdom; VetPath Specialists, Attica, Greece; VetPath Specialists, Attica, Greece; The Royal (Dick) School of Veterinary Studies, The University of Edinburgh and The Roslin Institute, Midlothian, United Kingdom; Department of Veterinary Anatomy, Physiology and Pathology, Institute of Infection, Veterinary and Ecological Sciences, University of Liverpool, Neston, United Kingdom; VetPath Specialists, Attica, Greece

**Keywords:** canine, Cushing’s syndrome, endocrinology, hormone, hypoadrenocorticism

## Abstract

**Background:**

In 2020, a permanent change in the antibody used in the Siemens Immulite 2000 cortisol assay resulted in a negative bias of approximately 23%. In response, the manufacturer introduced a veterinary-specific cortisol assay.

**Hypothesis/Objectives:**

To compare the analytical and diagnostic agreement between the Siemens Immulite 2000 standard cortisol (SC) and veterinary cortisol (VC) assays.

**Animals:**

Residual canine serum samples submitted to a veterinary diagnostic laboratory.

**Methods:**

Cortisol concentrations were measured using both SC and VC kits on the Siemens Immulite 2000 chemiluminescent immunoassay analyzer.

**Results:**

A total of 105 dogs contributed 154 cortisol measurements. Median (minimum, maximum) cortisol concentrations for VC and SC assays were 4.19 and 4.67 (<1.00, > 50.00) μg/dL, respectively. Further analysis included only 1 measurement per dog. Cortisol concentrations measured by the 2 assays were highly correlated (Spearman’s rho = 0.981, *P* < .001). Passing–Bablok regression demonstrated no significant constant bias (intercept −0.035; 95% CI, −0.184 to 0.061) or proportional bias (slope 1.036; 95% CI, 0.997-1.098). Bland–Altman analysis showed no significant mean bias between assays (−0.494 μg/dL; 95% CI, −3.524 μg/dL to 4.513 μg/dL), with limits of agreement of −4.353 μg/dL to 5.342 μg/dL. Diagnostic agreement was excellent (Cohen’s κ = 0.961); 3/105 cases were classified differently, with discordant results occurring near diagnostic decision thresholds.

**Conclusions and clinical importance:**

The SC and VC assays demonstrate strong analytical and diagnostic agreement in canine serum. Use of either assay is unlikely to alter clinical interpretation in most cases, and both assays appear to share similar analytical characteristics.

## Introduction

Accurate measurement of serum cortisol is fundamental to the diagnosis and management of canine adrenal disorders, particularly hypoadrenocorticism and Cushing’s syndrome. Serum cortisol concentration is used to determine if additional dynamic testing for hypoadrenocorticism is warranted and for interpretation of dynamic endocrine tests; clinical decision-making often depends on a common established diagnostic threshold. Consequently, analytical accuracy and consistency of cortisol assays are critical for appropriate interpretation of results.^1-3^

Several methods have been validated for measuring serum cortisol in dogs, including radioimmunoassays, chemiluminescent assays, and ELISA.^4-9^ The Siemens Immulite 2000/2000 XPi chemiluminescent immunoassay platform is widely used for cortisol measurement in veterinary diagnostic laboratories. As with other immunoassays, analytical performance is influenced by antibody specificity, cross-reactivity, and matrix effects. Additionally, assay imprecision has been reported to be greater at lower cortisol concentrations.^6^ These characteristics highlight the importance of ongoing analytical validation and quality assurance, particularly when methodological changes occur.^10^

In late 2020, a permanent change in the antibody used in the Siemens Immulite 2000 cortisol assay occurred, beginning with a new reagent lot series (lot 550). Data from the European Society of Veterinary Endocrinology (ESVE) quality assurance scheme demonstrated a negative bias in canine serum cortisol concentrations after this change, with an average bias of −23% compared with historical results, and an even larger negative bias reported in canine urine cortisol measurements.^11^ This bias was not constant across the analytical range, raising concerns that results near established clinical decision thresholds could be disproportionately affected, with potential implications for diagnosis and monitoring of diseases associated with abnormal cortisol concentrations.^11^

In response to the identified bias, the manufacturer introduced a veterinary-specific cortisol assay on the Immulite 2000/2000 XPi platform (“veterinary cortisol”) intended to improve alignment of canine cortisol results and mitigate assay differences. However, despite its widespread clinical adoption, an independent analytical evaluation directly comparing the standard cortisol assay and the veterinary cortisol assay on this platform has not, to our knowledge, been published. Moreover, it remains unclear whether any analytical differences between these assays translate into clinically important differences in diagnostic classification of canine cases.

The objectives of the present study were to: (1) compare the analytical agreement between the Siemens Immulite 2000 standard cortisol assay and the Siemens Immulite 2000 veterinary cortisol assay in canine serum; and (2) evaluate whether any observed analytical differences between assays result in differences in clinical classification of dogs investigated for hypoadrenocorticism or Cushing’s syndrome, or monitored during treatment of Cushing’s syndrome.

## Materials and methods

This was a retrospective method-comparison study. Residual serum samples from dogs submitted to a commercial veterinary diagnostic laboratory were used in our study. Samples were originally collected for clinical purposes unrelated to this study, including investigation of suspected hypoadrenocorticism or Cushing’s syndrome and monitoring of dogs undergoing treatment for Cushing’s syndrome.

Samples were eligible for inclusion if sufficient residual serum volume was available to allow cortisol measurement and if the reason for measuring serum cortisol concentration was known. No additional blood sampling was performed for the purposes of this study. All samples were anonymized before analysis. No animal- or owner-identifying information was retained.

Cortisol concentrations were measured using the Siemens Immulite 2000 chemiluminescent immunoassay analyzer (Siemens Healthcare Diagnostics, Germany). Each sample was measured using 2 commercial kits, namely, the standard cortisol kit (lot number 633) and the veterinary cortisol kit (lot number 049). Both assays were performed in accordance with the manufacturer’s instructions. Calibration and internal quality control procedures followed routine laboratory protocols. Both cortisol assays were performed on the same day and within the same run for each sample to minimize analytical variability. Internal quality control materials supplied by the manufacturer were analyzed according to routine laboratory schedules. Quality control results were reviewed by a board-certified clinical pathologist to ensure assay performance was within acceptable limits before inclusion of results in the analysis.

To evaluate analytical imprecision of both assays, pooled canine serum samples with low and high cortisol concentrations representative of clinically relevant decision levels (1 and 17 μg/dL) were analyzed. Within-run imprecision was assessed by measuring each pooled sample 10 consecutive times within a single analytical run for both the standard cortisol and veterinary cortisol assays. Between-run imprecision was assessed by measuring the same pooled samples across 10 separate analytical runs. Coefficients of variation (CVs) were calculated for both within-run and between-run imprecision for each assay.

Data distribution was assessed for normality using the Shapiro–Wilk test. Descriptive statistics were calculated. Correlation between cortisol concentrations measured by the 2 assays was assessed using the Spearman’s correlation coefficient, after the assessment of data distribution. Passing–Bablok regression analysis and Bland–Altman analysis were performed to evaluate the analytical agreement between the standard cortisol assay and the veterinary cortisol assay, with the latter designated as the reference method for the purposes of our study. For dogs with more than 1 cortisol measurement due to ongoing dynamic endocrine testing, a single measurement per dog was included in the correlation, Passing–Bablok regression, and Bland–Altman analyses to ensure independence of observations. To explore potential clinical impact, samples were classified according to clinical decision thresholds for screening or diagnosis of hypoadrenocorticism and investigation or monitoring of Cushing’s syndrome. Agreement in classification between assays was evaluated descriptively and using the Cohen’s κ value. Statistical analyses were performed using R statistical language (R Foundation for Statistical Computing, Vienna, Austria). A significance level of 0.05 was used for all analyses.

## Results

One hundred and five dogs contributing a total of 154 cortisol measurements were included in this study. Median (range) age was 11 (1-17) years, with 55 female and 50 male dogs. Serum cortisol concentration was measured in 52/105 (47.6%) dogs to determine if further dynamic testing was warranted for possible hypoadrenocorticism. Samples were submitted after an ACTH stimulation test in 36/105 (34.3%) dogs (17/36 [47.2%] for diagnosis of Cushing’s syndrome, 14/36 [38.9%] for diagnosis of hypoadrenocorticism, and 5/36 [13.9%] for monitoring of response to treatment of Cushing’s syndrome). In 11/105 (10.5%) dogs, samples were submitted after a low-dose dexamethasone suppression test (LDDST) and in 5/105 (4.8%) dogs a pre-pill cortisol was measured for monitoring of Cushing’s syndrome.

**Figure 1:**
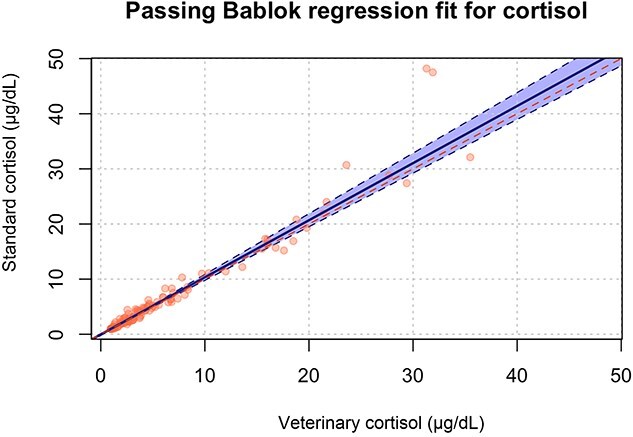
Passing–Bablok regression analysis of serum cortisol concentration measured using the Siemens Immulite 2000 standard cortisol assay compared with the Siemens Immulite 2000 veterinary cortisol assay in 105 serum samples from dogs. The red diagonal line is the line of identity and the blue line is the calculated line of regression. The light blue area represents the 95% CIs.

**Figure 2:**
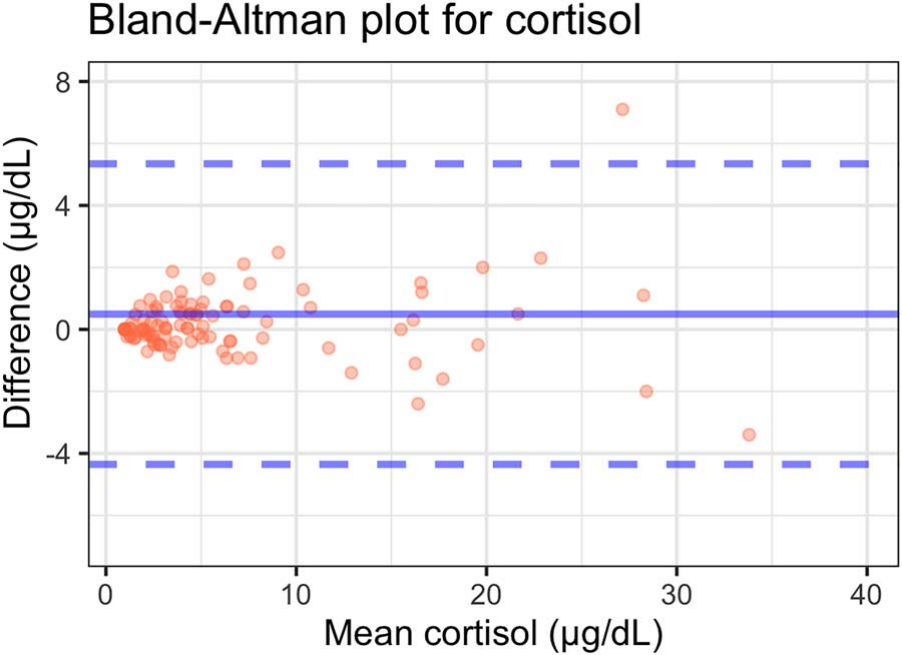
Bland–Altman analysis of serum cortisol concentration measured using the Siemens Immulite 2000 standard cortisol assay compared with the Siemens Immulite 2000 veterinary cortisol assay in 105 serum samples from dogs. The difference between the 0 line and the blue line indicates the bias of the standard cortisol assay minus the veterinary cortisol assay. The 95% CIs of the calculated bias are represented by the 2 blue dashed lines.

When all measurements were taken into consideration, the median cortisol concentration measured by the veterinary assay was 4.19 (<1.00 to > 50.00) μg/dL and the median cortisol concentration measured by the standard assay was 4.67 (<1.00 to > 50.00) μg/dL. When only one measurement per dog was included, the median cortisol concentration was 3.84 μg/dL (range, < 1.00-35.50) for the veterinary assay and 4.30 μg/dL (range, < 1.00-48.20) for the standard assay. A strong positive correlation was observed between cortisol concentrations measured using the 2 assays (ρ = 0.981, *P* < .001). Passing–Bablok analysis identified no significant constant bias (intercept: −0.035; 95% CI, −0.184 to 0.061) or proportional bias (slope: 1.036; 95% CI, 0.997-1.098) (Figure 1). Bland–Altman analysis revealed no statistically significant mean bias between assays (0.494 μg/dL; 95% CI, −3.524 μg/dL to 4.513 μg/dL). The lower and upper limits of agreement were −4.353 μg/dL (95% CI, −5.182 μg/dL to 0.973 μg/dL) and 5.342 μg/dL (95% CI, 0.015 μg/dL to 6.171 μg/dL), respectively (Figure 2).

Analytical imprecision of both assays was evaluated using pooled canine serum samples with low and high cortisol concentrations. Mean cortisol concentrations of the pooled samples were 1.93 and 16.65 μg/dL for the standard cortisol assay and 1.96 and 16.44 μg/dL for the veterinary cortisol assay. The within-run CV for the standard cortisol assay was 5.8% and 5.2% at low and high concentrations, respectively, and 5.7% and 5.1% for the veterinary cortisol assay. Between-run imprecision CV for the standard cortisol assay was 7.0% and 9.0%, and for the veterinary cortisol assay 6.8% and 9.5% at low and high concentrations, respectively. The Cohen’s κ value was 0.961, with 3/105 dogs being classified differently. In 2 dogs tested for basal cortisol to rule out hypoadrenocorticism, veterinary cortisol was 1.84 μg/dL (in both dogs) and standard cortisol was 2.14 and 2.80 μg/dL, resulting in discordant interpretation when using the common cut-off value of 2 μg/dL. In the third dog, tested for Cushing’s syndrome using a LDDST, veterinary cortisol concentrations at 0, 4, and 8 h (2.15, 1.46, and 1.15 μg/dL, respectively) suggested lack of suppression, whereas standard cortisol concentrations (1.97, 1.21, and < 1.00 μg/dL, respectively) suggested suppression at 8 h.

## Discussion

In this method-comparison study, the Siemens Immulite 2000 standard cortisol assay and the veterinary cortisol assay demonstrated strong analytical agreement when measuring cortisol in canine serum. No significant constant or proportional bias was identified, and overall agreement across the studied concentrations was strong. These findings indicate that, under the conditions of this study, the 2 assays performed similarly when applied to routine diagnostic samples from dogs. Consistent with this, clinical classification was concordant in 97% of cases, with discordant results observed at cortisol concentrations near commonly used diagnostic thresholds.

There was an excellent correlation between the 2 assays. Passing–Bablok regression analysis demonstrated a strong linear association between cortisol measurements obtained by the 2 assays (Figure 1). The estimated intercept (−0.029; 95% CI, −0.155 to 0.030) included zero, indicating no evidence of constant bias, while the slope (1.029; 95% CI, 0.987-1.078) included unity, indicating no evidence of proportional bias across the analytical range. Although visual inspection of the Passing–Bablok plot suggested a slight deviation from the line of identity at higher cortisol concentrations, this deviation was not supported by the confidence intervals of the regression parameters and therefore does not represent statistically significant proportional bias in the present dataset. However, the number of samples with very high cortisol concentrations was limited, and a proportional positive bias at the upper end of the analytical range cannot be excluded.

Bland–Altman analysis showed a small positive mean difference between methods (0.4 μg/dL), with the 95% CI including zero (−3.8 μg/dL to 4.6 μg/dL), indicating no statistically significant systematic bias (Figure 2). The limits of agreement were narrow (−3.2 μg/dL to 4.0 μg/dL), with corresponding confidence intervals indicating reasonable precision of estimation. Visual inspection of the Bland–Altman plot suggested a mild concentration-dependent pattern, with slightly greater dispersion at higher mean cortisol values. However, this pattern was not corroborated by the CIs around the mean bias or the limits of agreement, indicating that the apparent trend could reflect random variability rather than true proportional bias. As with the Passing–Bablok analysis, the number of samples with high cortisol concentrations was limited, and therefore the presence of proportional bias at the upper end of the analytical range cannot be completely excluded. Under the conditions of this study, taken together, Passing–Bablok and Bland–Altman analyses indicate good analytical agreement between the 2 cortisol assays, with no statistically significant constant or proportional bias, supporting their comparability within the investigated concentration range.

Agreement in clinical classification between assays was high, with 3 of 105 (2.9%) cases classified differently by the 2 assays. Of these, 2 discordant classifications occurred at cortisol concentrations close to commonly used diagnostic thresholds (1 and 2 μg/dL), with values falling just below the cut-off using the veterinary assay and just above the cut-off using the standard cortisol assay. Interpretation of cortisol concentrations near diagnostic decision thresholds should also take into account analytical variation. When bias is negligible (as here), expanded measurement uncertainty can be approximated as 2 times the analytical CV, providing an estimate of the range within which the true value is expected to lie.^12^ Using the between-run CV calculated in this study (7%), the expanded uncertainty around a result of 2 μg/dL would be approximately ± 0.28 μg/dL, and for a result of 1 μg/dL approximately ± 0.14 μg/dL. These ranges overlap clinically relevant decision thresholds and therefore could account for the observed discordant classifications between assays. This supports the interpretation that such discordant results are unlikely to represent true biological differences but rather reflect expected analytical imprecision near decision limits.

From a practical perspective, these findings are reassuring for clinicians and clinical pathologists, as they suggest that use of either assay is unlikely to result in differences in clinical interpretation in most cases. Consideration of cost-effectiveness is relevant in veterinary diagnostics, especially when analytical performance between assays appears equivalent^10^; this is an important consideration in the present context, as the cost of the standard cortisol assay kit is substantially lower than that of the veterinary cortisol kit. Based on the results of the present study, routine preference for the veterinary cortisol assay over the standard cortisol assay does not appear justified based solely on analytical agreement in this dataset.

At the same time, the findings must be interpreted in the context of the assay changes reported in 2020. The veterinary cortisol assay was introduced in response to a documented substantial negative bias associated with an antibody change in the standard Immulite 2000 cortisol assay.^11^ The analytical similarity observed in the present study suggests that the veterinary cortisol assay has not effectively addressed that earlier bias and it remains possible that both assays share similar analytical limitations relative to pre-2020 performance or to reference methods. *Importantly, the present study evaluated agreement between the 2 Siemens Immulite 2000 assays rather than absolute accuracy compared to a reference standard.* Previous veterinary endocrinology studies have emphasized the importance of comparison with reference methods, such as radioimmunoassay (RIA), when evaluating cortisol immunoassays, antibody specificity, and cross-reactivity.^8^  *Therefore, conclusions regarding the trueness of either assay relative to a reference method cannot be drawn.*

A limitation of this study is that comparison with a reference method was not performed, precluding assessment of absolute analytical accuracy or direct evaluation of whether the veterinary cortisol assay mitigates the negative bias previously reported. In addition, samples were not measured in duplicate using both assays, as ideally recommended for method comparison studies.^13^ However, duplicate measurements were not feasible because only surplus serum samples were available and sample volume was insufficient to permit 4 cortisol measurements per specimen in most samples. A third limitation of the present study is that only a limited number of samples with high cortisol concentrations were included; therefore, the absence of proportional bias at the upper end of the analytical range cannot be confirmed with certainty. Nonetheless, this reflects the relative rarity of such samples in clinical practice.

In conclusion, under the conditions of our study, the Siemens Immulite 2000 standard cortisol and veterinary cortisol assays demonstrate strong analytical agreement in canine serum. While this finding supports consistency in clinical interpretation, it also suggests that the veterinary cortisol assay might not confer substantial additional analytical benefit compared with the standard cortisol assay, particularly when considering the substantially higher cost of the veterinary cortisol kit. Further investigation using a reference method, such as a validated RIA assay, is required to assess absolute analytical accuracy, clarify the effect of previous assay modifications, and define the optimal approach to cortisol measurement in dogs.

## Data Availability

Data are available upon request from the first author.

## Off-label antimicrobial declaration

The authors declare no off-label use of antimicrobials.

## Institutional animal care and use committee or other approval declaration

Ethical approval was not required for this study, as only residual, anonymized serum samples collected for routine diagnostic purposes were used, and no additional procedures were performed on animals for research.

## Abbreviations

CV: coefficient of variation
ESVE: European Society of Veterinary Endocrinology
RIA: radioimmunoassay
SC: standard cortisol
VC: veterinary cortisol
